# The Electrodegradation Process in PZT Ceramics under Exposure to Cosmic Environmental Conditions

**DOI:** 10.3390/molecules28093652

**Published:** 2023-04-22

**Authors:** Iwona Lazar, Christian Rodenbücher, Gustav Bihlmayer, Clive A. Randall, Janusz Koperski, Lutz Nielen, Krystian Roleder, Krzysztof Szot

**Affiliations:** 1 August Chełkowski Institute of Physics, University of Silesia in Katowice, ul. 75 Pułku Piechoty 1, 41-500 Chorzów, Poland; iwona.lazar@us.edu.pl (I.L.); janusz.koperski@us.edu.pl (J.K.); krzysztof.szot@us.edu.pl (K.S.); 2 Forschungszentrum Jülich, Institute of Energy and Climate Research (IEK-14), 52425 Jülich, Germany; c.rodenbuecher@fz-juelich.de; 3 Peter Grünberg Institut and Institute for Advanced Simulation, Forschungszentrum Jülich and JARA, 52425 Jülich, Germany; g.bihlmayer@fz-juelich.de; 4 Center for Dielectrics and Piezoelectrics, Materials Research Institute, Department of Materials Science and Engineering, The Pennsylvania State University, University Park, PA 16802, USA; car4@psu.edu; 5 aixACCT Systems GmbH, 52068 Aachen, Germany; nielen@aixacct.com

**Keywords:** PZT ceramics, electrodegradation, oxygen vacancies, oxygen effusion, grain boundaries, dislocations, DFT calculations

## Abstract

Long-time electric field action on perovskite piezoelectric ceramic leads to chemical degradation. A new way to accelerate the degradation is the exposure of the ceramic to DC electric fields under a vacuum. A high-quality commercial piezoelectric material based on PbZr_1−x_Ti_x_O_3_ is used to study such impacts. To avoid the influence of ferroelectric properties and possible removal of oxygen and lead oxides during the degradation process, the experiments are in the temperature interval of 500 °C > T > T_C_. Changes in resistance during the electrodegradation process is an electrically-induced deoxidation, transforming the ceramic into a metallic-like material. This occurs with an extremely low concentration of effused oxygen of 10^16^ oxygen atoms per 1 cm^3^. Due to this concentration not obeying the Mott criterion for an isolator-metal transition, it is stated that the removal of oxygen mostly occurs along the grain boundaries. It agrees with the first-principle calculations regarding dislocations with oxygen vacancies. The decrease in resistivity during electrodegradation follows a power law and is associated with a decrease in the dislocation dimension. The observed reoxidation process is a lifeline for the reconstructing (self-healing) properties of electro-degraded ceramics in harsh cosmic conditions. Based on all of these investigations, a macroscopic and nanoscopic model of the electrodegradation is presented.

## 1. Introduction

Extreme environmental operating conditions for piezoelectric sensors or actuators for geological [1], industrial, medical [2], military [3], and other applications have been achieved, for example, under high temperatures, with high mechanical–electrical loads and/or humidity conditions [4,5]. However, the design of piezoelectric sensors for space technology must consider electrodegradation under vacuum conditions [6,7,8,9]. The literature presents different models of how the piezoelectric, physical, and chemical properties change due to fatigue and degradation effects [10,11,12,13,14,15,16,17,18]. Electric breakdown by direct current (DC) degradation [19,20] and resistance switching [21,22] belongs to these effects. However, direct analysis of the stability and reliability of piezoelectrics in a cosmic vacuum has not yet been openly reported. This work aims to determine the nature of the degradation of the most widely used piezoelectric PbZr_1−x_Ti_x_O_3_ (PZT) ceramics (here, the commercial PIC 151, produced by PI) caused by low oxygen activity, typical under cosmic conditions and high DC electric biases. We have considered many models that describe the physics and chemistry of the fatigue or degradation effect in piezo-ceramics [23,24,25,26,27] for defining the boundary conditions for our experiments. According to point defect chemistry [28,29,30,31,32,33], in temperatures lower than 500 °C, oxygen or lead vacancies’ thermal generation can be neglected. Therefore, this process cannot contribute to the degradation processes of ceramics. It is noteworthy that others have identified similar conditions for this kind of investigation [26,27].

The best approach to describe the kinetics of electrodegradation requires the time-dependence of the potential distribution to be investigated [20,34,35,36,37,38,39,40]. For this, we developed a unique electrometric high-voltage multi-electrode system. It is important to recall that electrical stimuli could induce stoichiometric polarisation [24,25] within the piezoelectric materials, which can, in turn, change local ceramics’ compositions. Therefore, an essential consideration of our investigation is the control of local stoichiometry deviations, especially in the vicinity of electrodes. Studies using optical microscopy allow the identification of localised discoloured regions in electro-degraded ceramics [41,42]. Analysing physical and chemical properties on the macro- and nano-scales of well-defined crystalline boundaries, as in bi/crystalline boundaries, has been shown to be extremely informative [41,42], and similar experiments have targeted the grain boundaries in PZT ceramics. Using the etching technique, we analysed the distribution of etch pits marking the points of dislocation in stoichiometric and electro-degraded regions on metallographically-polished ceramics. Finally, the ab initio calculations of the electronic structure of dislocations as conducting filaments in different defect states were undertaken.

The degradation process induced by the DC acting on ceramics was often associated with the electromigration of the cations from the electrodes, such as Ag and Cu [27,43,44,45]. In order to avoid the influence of dendrite formation on electrode materials along the grain boundaries, platinum was used as the electrode material. In addition to researching the effect of an electric field on solid ceramics, polycrystalline thin films were investigated to analyse electrodegradation at the nano-scale.

## 2. Results

### 2.1. Electrically-Stressed PZT Ceramics under Vacuum Conditions at an Elevated Temperature (T > T_C_)

**Figure 1:**
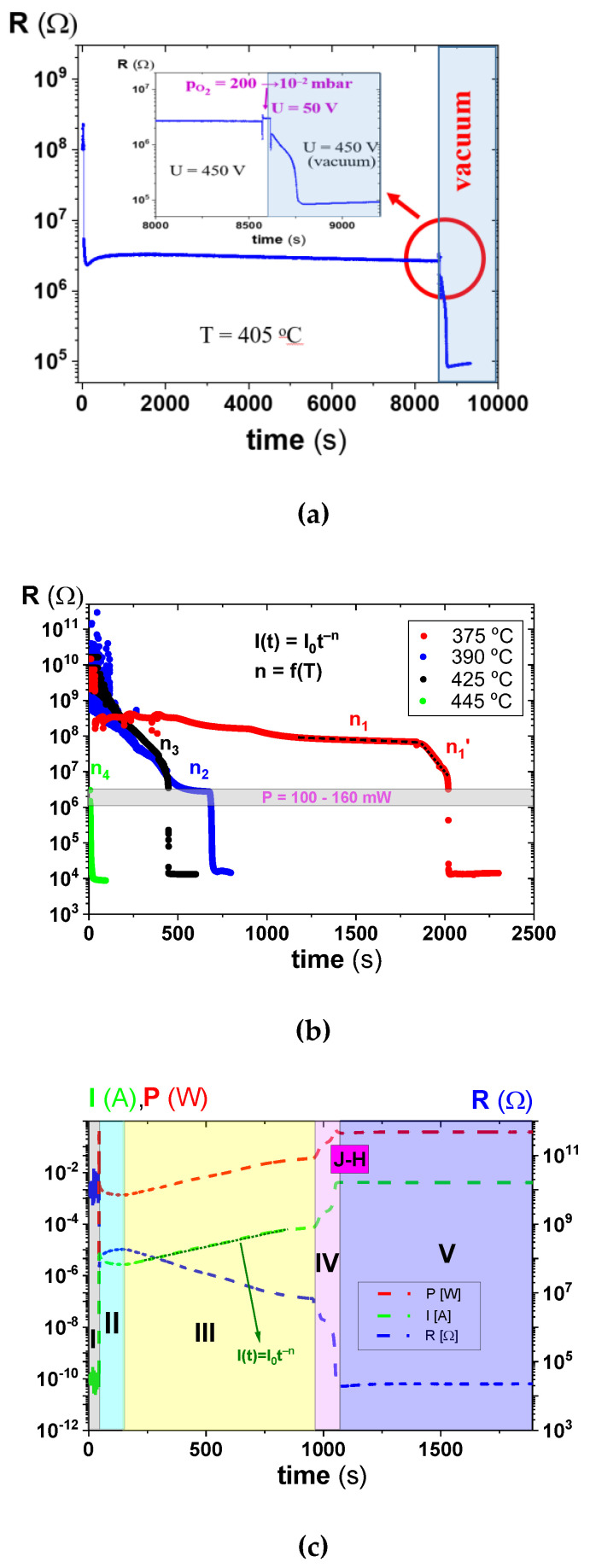
Electro-reduction of the PZT (PIC 151) ceramics at U_max_ = 450 V and under a vacuum to simulate space conditions. (**a**) The vacuum caused the electrodegradation process to progress rapidly. The inset shows an enlargement of the electrodegradation curve in which the acceleration of the resistance reduction (the section marked with a red circle) becomes visible; (**b**) the shortening of the times for the complete electrodegradation of the ceramics when the temperature increases display an activating character in the process. The power law I(t) with a parameter n depends on the temperature [5]; and (**c**) there is a decrease in the resistance of the ceramics by several orders of magnitude, which occurs over five steps, labeled from I to V (see text for detailed description).

The purpose of this study was to investigate the electrodegradation process that occurs when an electric field is applied to ferroelectric ceramics. The research concerns PZT ceramics used in electronic devices under reduced pressure conditions. An example of such a process, which proceeds similarly to other binary and ternary transition metal oxides [22,41,45], is presented in Figure 1. Although normal conditions of long-term field action do not cause significant changes in resistance, reducing the pressure around the sample causes this process to accelerate significantly. The course of the electrodegradation process, represented in Figure 1c, can be divided into several characteristic stages:

1. Stage I—A steep step-like increase of the current flow (green curve in Figure 1c) due to displacement and Ohmic currents after a high voltage is switched on;
2. Stage II—Decreasing the electric current due to discharging of the capacitor; only the Ohmic contribution of the so-called leakage current contributing to the total current remains;
3. Stage III—The resistance (blue line in Figure 1c) shows an exponential decrease with time; the slope of the log(R(t)) curve increases with increasing temperature (see Figure 1b);
4. Stage IV—A dramatic increase of the kinetics of the electrodegradation; positive feedback from a Joule heating (J-H peak in Figure 1c) is presented. In this way, in a short time, the resistance of the sample is strongly reduced. The sample temperature can increase in this phase by about 10–20 °C [42];
5. Stage V—In this final stage, the electrical decomposition process stagnates, and the resistance change is minimal because, in the current flow, the transport of electrons dominates, and the ionic transport, which is responsible for reducing the oxygen stoichiometry in the ceramic, does not play an important role.

The effect of such a process on dielectric properties is presented in Supplement Material S1, Figure S1c.

The electrodegradation process is not only time-dependent, but its rate also depends on sample temperatures. The overall collection of the electrodegradation curves measured under various temperatures indicates that resistance degradation and oxygen activity show a dramatic acceleration (Figure 1b) when temperature increases. This means that the process is thermally activated. Only stage IV is similar across different temperatures, namely at the start of the sudden increase in the progression of electrodegradation. This behaviour is observed for a specific resistance (3–5 × 10^6^ Ω), which therefore gives a power loss limit for the acceleration of the electrodegradation for all samples of several hundred mW (Figure 1b). From the samples with the same geometry and applied electrical field stress, the positive thermal feedback is related to Joule heating once the power loss has reached a specific level and leads to the step-like change in the resistance.

**Figure 2:**
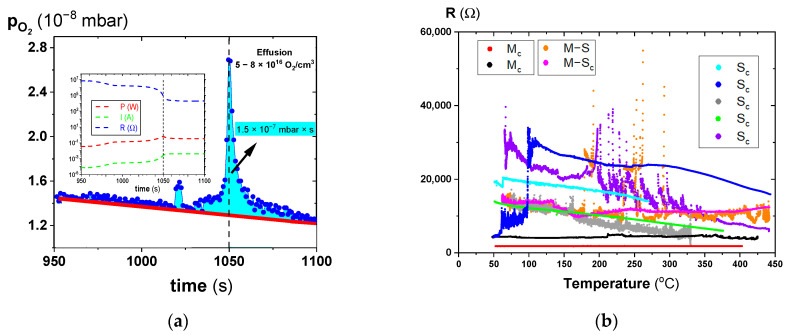
(**a**) Oxygen effusion near the region with the strongest progression of the process; the inset depicts the simultaneous measurement of resistance R, current I, and power dissipation P; and (**b**) resistance-dependence of the PZT ceramics for different electrodegradation parameters. Three resistance characteristics were observed as a function of the temperature: M_c_ marks curves with metallic character, the M_c_-S_c_ corresponds to mixed dependence, high-temperature metallic and low-temperature semiconducting, whereas the S_c_ curves are thermal dependencies of the resistance with shallow activation energy (20 meV) after the electrodegradation process.

The simultaneous measurements of the electrical parameters (inset of Figure 2a) and the effusion of gases (Figure 2a) during the electrodegradation show that the system cannot be analysed as a closed (isolated) one. During step IV (at around 1050 s of measurement), a dramatic rise in oxygen effusion from the specimen was identified and quantified using the QMS spectrometer. The transference number is determined by comparing the total electronic and ionic conductivity, which was equal to 0.03. This means that no considerable amount of oxygen escapes from the sample volume during the electrodegradation process. Electrically-induced deoxidation is responsible for incorporating oxygen vacancies into the system, i.e., removing oxygen from the sample. As a result, a reduction occurs in the oxidation state of the transition metal ions (here Ti^4+^→Ti^3+^/Ti^2+^ and Zr^4+^→Zr^3+^/Zr^2+^); this is the origin of what we call electrically-induced “n-type” doping. Although the oxygen outflow is extremely low at only 10^16^ molecules per 1 cm^3^ (this concentration was calculated assuming the entire sample was homogeneously electro-degraded), the ceramic was transformed into a metallic object (Figure 2b).

The evidence of oxygen effusion allows the electroreduction to be classified as a typical reduction reaction (deoxidation) consistent with point defect chemistry (see Equation (1) for the removal of oxygen in Kroeger–Vink notation [46]):

(1) $$O_{O}\to Vö+2e^{\prime }+\frac{1}{2}O_{2}↑$$

The origin of this effusion cannot be associated with the classical thermal activation process from the volume, as point defect chemistry for temperatures below 500 °C does not predict oxygen exchange between the ambient environment and the entire sample [47]. In contrast to the exponential decrease in the resistance for the stoichiometric ceramics with increasing temperature [5], the electro-degraded sample shows a metallic-like conduction character with extremely low activation energy (20 meV). This activation energy level reflects a radical change in conduction and is in the range of polaron and related hopping electron transport [33,48,49,50]. In particular, the transformation of the insulating ceramic sample to the metallic state by electrodegradation after the removal of only 8 × 10^16^/cm^3^ oxygen atoms (see Figure 2a) underlies a sophisticated analysis of the nature of this transition. For the isolator-metal (I/M) transition in transition metal oxides (e.g., for model materials with perovskite structure—SrTiO_3_), according to the Mott criterion, it is necessary to dope an oxide system with at least 5 × 10^18^/cm^3^ charge carriers [20]. In our case, the carriers are the d^1^ electrons coming from reduced Ti or Zr ions as an effect of creating oxygen vacancies near the mentioned ions. However, the concentration of oxygen vacancies is two orders of magnitude lower than necessary for Mott’s criterion. Only when assuming unrealistic values of the effective mass of d^1^ electrons and the dielectric coefficient of electro-reduced PZT could the criterion be valid for uniformly-distributed charge carriers. It is worth mentioning here that aliovalent doping with Nb up to a concentration of 7% mol does not lead to a I/M transition [51]. Moreover, it is worth noting that from the dependencies of R(T) (Figure 2b), which show the mixed metallic and semiconducting conductivity with very low activation energy, a form of temporal change between metallic and semiconducting states can be observed. This means that this electrical state, caused by incomplete electrodegradation, is metastable. On the energetic scale, the two states are “separated” with a minimal barrier (see, e.g., the DFT analysis for electro-degraded SrTiO_3_ crystals) [20]. It is also noteworthy that the heavily donor-doped oxide ferroelectrics experience a perturbation in the Mott criterion and so the latter should be considered a guide, but could have departures in the ferroelectric phase measurements below T_C_ [33,48,49,50].

### 2.2. Oxidation of Electro-Degraded Ceramics. Reversibility of I/M Transition

**Figure 3:**
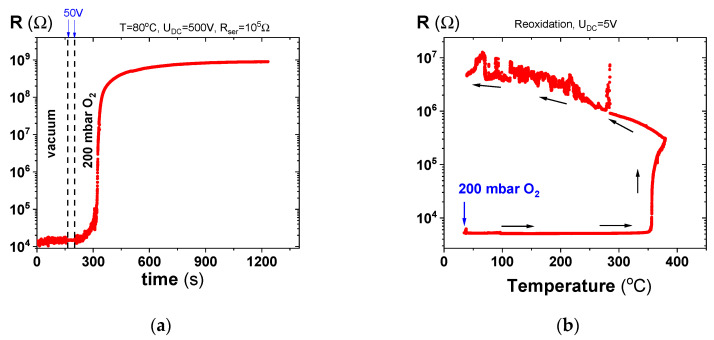
(**a**) Resistance of electro-degraded PZT measured during the sudden change in oxygen activity, from the resting pressure of oxygen in a vacuum to 200 mbar at 80 °C when a voltage of 500 V was applied. Note that the voltage was reduced to 50 V during the pressure change to avoid glow discharge; and (**b**) resistance as a function of temperature during exposure of an electro-degraded sample to 200 mbar of oxygen when applying a low voltage only.

Here, we consider the possibility of a “switch” back into an insulated state by a reoxidation process. We have investigated this effect of restoration of insulating properties following the electrodegradation of PZT ceramics for two different oxidation conditions, as shown in Figure 3. Our test confirmed that for both conditions, it is possible to restore the insulating properties. Not surprisingly, electrochemically-forced oxidation (Figure 3a) works more effectively than oxygen incorporation by a chemical gradient alone, i.e., a high oxygen pressure in this case (Figure 3b).

This reversibility of the electrochemical degradation process provides insights into possible countermeasures that would limit the electrodegradation processes under space environmental conditions. For example, a PZT specimen should be hermetically sealed in a box with higher oxygen pressure—they are already used in the case of actuators working in ultra-high vacuum (UHV) environments of up to 10^−10^ mbar or coated with an ionic oxide as a source of oxygen to limit electrodegradation of PZT through deoxidation.

### 2.3. Inhomogeneous Electrodegradation. Raman Scattering Analysis of the Electro-Degraded Ceramics

As mentioned above, based on the effusion data (Figure 2a), the calculated concentration of oxygen vacancies is 2.5 orders of magnitude lower than the Mott criterion. Considering the low average concentration of oxygen vacancies, it is a feasible hypothesis that the Mott criterion is fulfilled only locally or, in other words, that the electrodegradation results in the formation of conducting filaments. Under this scenario, the local regions with metallic-like conduction should be galvanically interconnected to each other to allow the macroscopic transition of the ceramic to a metallic-like conduction state, but still with most of the PZT microstructure remaining in an original stoichiometric and insulating condition.

**Figure 4:**
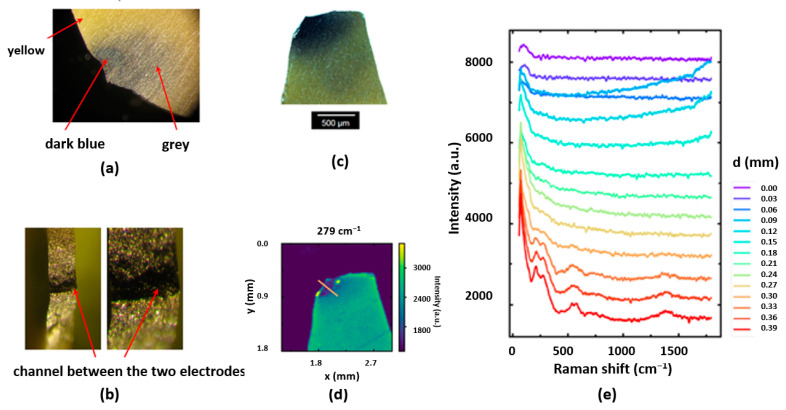
Optical inspection of the electro-degraded ceramics. (**a**) Surface after removal of the electrode; after electrodegradation, it has become coloured, from yellow to dark blue and grey; (**b**) overview and cross-section; the dark part is limited to a small channel between the two electrodes; (**c**) Raman studies of the electro-degraded PZT ceramics—optical microscopy image; (**d**) intensity of the Raman line at 279 cm^−1^ corresponding to ZrO_3_ torsional modes; and (**e**) Raman spectra for different sample positions along the line marked in (**d**) show distinct changes in the intensity of the central peak and the 200–500 cm^−1^ range.

As reduction through the electrodegradation of perovskite oxides lead to the introduction of d^1^ electrons, this also causes strong optical absorption [20]; the regions with a higher concentration of metallic d^1^ electrons of Ti and Zr can be then identified through an optical inspection. According to our analysis of the Mott criterion, we must assume that d^1^ electron doping is also non-homogeneous, which is confirmed by dark areas in the electro-degraded ceramics (Figure 4a–c). This visual analysis suggests that the oxygen vacancies generated in the sample, whose concentration equals the amount of effused oxygen, are only located in channels. However, the EDS mapping (Supplement Materials S1) shows no deviation from the correct chemical composition for different sample regions (Figure S1a,b). The remaining area of the sample retains its unchanged colour and, as we confirmed, shows no metallic conductivity.

In order to obtain a more detailed insight into the properties of the electro-reduced region, the same piece, which was investigated by optical microscopy, was analysed by Raman spectra-microscopy. Figure 4d depicts a map of the intensity in the wavenumber range between 269 cm^−1^ and 289 cm^−1^, where the Raman active E mode is located. It can be seen that the intensity close to the electro-degraded region is much lower than in the outer part of the sample. This is exemplified in Figure 4e, where Raman spectra at different positions along the line shown in Figure 4e are plotted. While the spectra obtained at the bottom right end of the line reveal a typical Raman spectrum of PZT, those recorded in the electro-degraded region show almost no characteristic Raman peaks. This indicates that the Raman effect in the electro-degraded region is suppressed, presumably because of a local increase in charge carrier density, as would be expected in the case of metallic-like behaviour, leading to a loss of polarizability.

According to Kozielski, et al. [52] in PZT, the E(TO2) mode describing vibrations of the Zr^4+^ and Ti^4+^ cations against the oxygen octahedra consists of two split modes in the range of 210–240 cm^−1^. The lowest frequency E(TO1) and A1(TO1) phonons in the spectra originate from Pb ions vibrating against Zr/TiO_6_ octahedra and correspond to the two components of the soft mode. This system creates the central mode by hopping off-site Pb atoms amongst available sites [53]. In the Raman spectrum of the yellow part of the ceramics, these peaks can be identified (red curve in Figure 4e). Close to the electro-reduced blackened parts, these peaks vanish, but no new ones can be detected, indicating that the transformation of the crystal changes mainly changes the electronic structure by inducing d^1^ states, but no evidence for a significant change in the crystallographic structure can be detected.

### 2.4. Resistance and Potential Distribution during Electrodegradation: The Deoxidation and Reoxidation Processes

**Figure 5:**
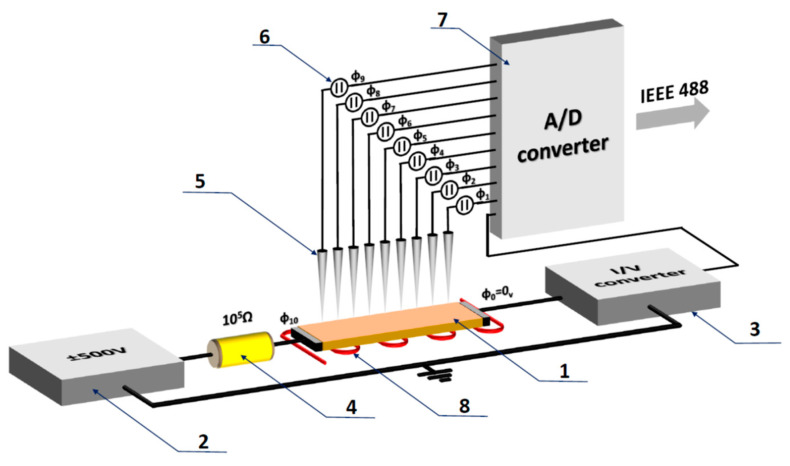
Schematic illustration of the measuring system for determining the potential distribution during electrodegradation. (1) Specimen with typical bar geometry (the Pt electrodes were deposited onto the smallest areas); (2) power amplifier/high voltage amplifier; (3) I/U converter with sensitivity from 10^−15^ A to 10^−1^ A; (4) series resistor automatically limits the maximum current (at 500 V) flowing through the sample in a metallic state to 5 mA; (5) sharp probes made of Pt/Ir alloys; (6) nine electrometers with reduced input capacitance (10^−15^ F) and input resistance of 10^14^ Ω. The electrometers (designed by aixACCT) can be used for voltages ± 1000 V; (7) 16-bit analogue-to-digital voltage converter; and (8) heating system RT—1000 °C. Note: The system operates in a vacuum chamber that can reach UHV conditions or 1000 mbar pressures of oxidising or reducing gases.

In order to investigate the electrodegradation’s spatial progression in detail, we chose the classical rod geometry for the PZT ceramic samples. A new Multi-Probes Analyser system (see Figure 5) was used to analyse the change in the potential distribution between the cathode and anode for each stage of electrodegradation (Figure 1).

The limitation of the electrodegradation study to stages III and IV (see Section 2.1) was connected to the reduction by ten orders of magnitude of the electrical field due to the ceramic’s bar length extension to 1 cm. A thin sample with capacitor geometry and a thickness of 1 mm could be easily fully degraded. Due to this, we can analyse the changes in different regions of the PZT ceramic using potential distribution for most essential stages of this process.

**Figure 6:**
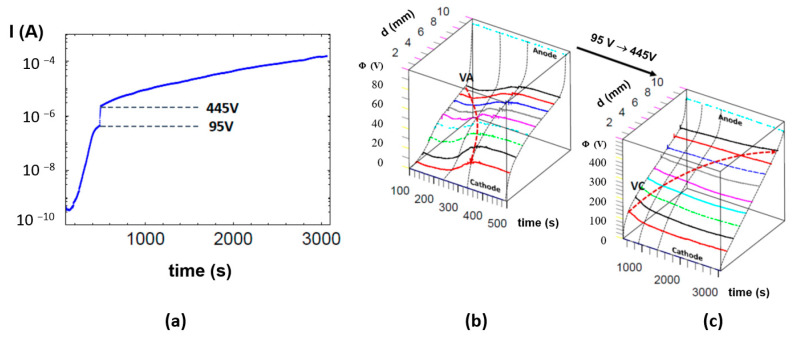
Time dependence of (**a**) the current, and (**b**,**c**) the potential distribution along the rectangular PZT ceramic sample (10 × 3 × 1 mm^3^) during the electrodegradation under simulated cosmic conditions (T = 440 °C, U = 95–445 V, R_serial_ = 10^5^ Ω). The nine sharp probes were positioned on the sample’s surface at an equidistance of 1 mm. The evolution of the local potential in different regions between the cathode and anode, detected by Multi-Probes Analyser, was shown for (**b**) low and (**c**) high voltage.

**Figure 7:**
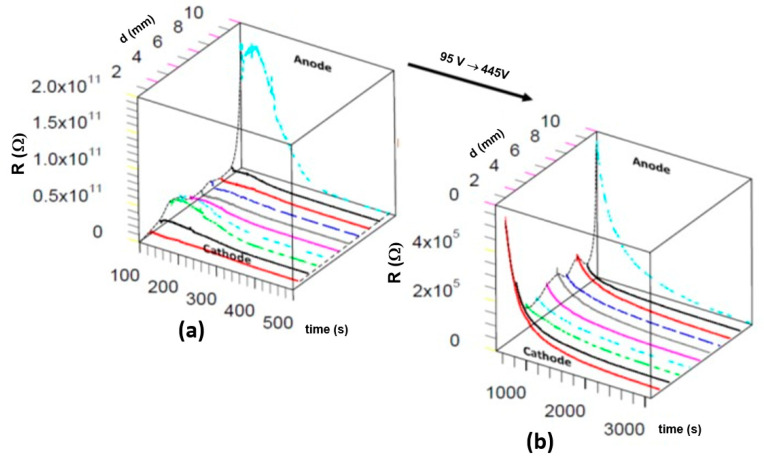
Local changes in resistance during the electrodegradation show that the regions close to the cathode and anode control the kinetic of the process for (**a**) a low voltage of 95 V, and (**b**) a high one of 445 V.

The simultaneous measurements of the overall resistance degradation (Figure 6a) and associated potential distribution (Figure 6b,c) open up a new possibility for the analysis of the contribution of different regions between the cathode and anode. After the voltage was switched on (Figure 6b), we observed that the region close to the anode controlled the overall current flow, in contrast to the region close to the cathode, which exhibited the lowest potential drop (Supplementary Material S2). The ceramic sample’s short-term voltage action in 150–200 s dramatically modified the potential distribution. Although the global resistance of the system Pt/PZT/Pt permanently decreased, the relative contributions to the resistance of the different regions changed during the electrodegradation time (Figure 7). It should be noted that we do not observe a monotonic change in the potential drops in all positions of the probes. The early stage of DC resistance degradation corresponds to the movement of the virtual anode (VA) towards the cathode (Figure 6b). The potential distribution took the form “S”, with the maximum potential drops close to the cathode and anode being similar; in this region, the local electric field reaches the maximum strength. In the last stage of the low-voltage action, the progression of the electrodegradation is slower (Figure 6a). After increasing the voltage from 95 to 455 V, the “S” type of potential distribution becomes semi-linearised, prolonging the electrodegradation time (Figure 6c). This transformation into a “semi-ohmic resistor” is connected to the reduction of the potential drop in the vicinity of the cathode. This behaviour is associated with the virtual cathode (VC) movement towards the anode.

The information presented enables analysis of the degradation from a new perspective. From the macroscopic two-point measurements, it can only be confirmed that the electrodegradation reduced the macroscopic resistance of the system Pt/PZT/Pt by 4–5 orders of magnitude. Using the measurement of the potential distribution, one can fully describe the contribution of each region to the reduction in the sample’s resistance. For electrodegradation with 95 V, the higher electric field is located near the anode and is ten times greater than that close to the cathode. At the end of this process, the difference in potential drops between the anode and cathode is only 2 (Figure S2a). A high voltage of 445 V leads to a more symmetrical potential distribution close to the electrodes (Figure S2b); the electric field in other regions is 3–4 times lower.

The general conclusion, formulated using the analysis of the local resistance change, seems trivial. For the low and high voltages, the reduction in the resistances of all regions progresses continuously with increasing degradation time. However, the relative decrease in resistance indicates that the maximum change occurs near the anode at the low voltage (Figure 7a). The enhancement of the voltage changes the character of the relative reduction in the resistance, especially in the vicinity of the cathode (Figure 7b).

**Figure 8:**
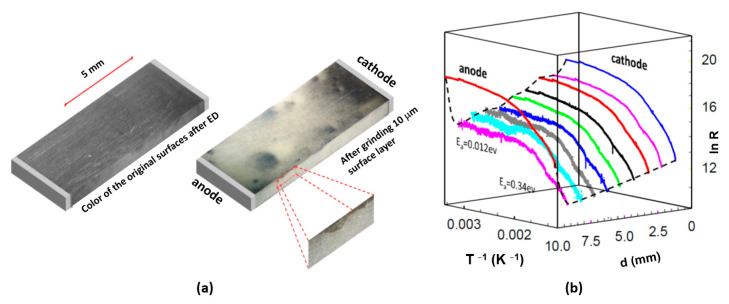
(**a**) Optical microscopy of the PZT ceramics after electrodegradation illustrated as a 3D figure. Left: original surfaces. Right: surface after mechanical removal of the 10 µm thick layers of the two largest surfaces; and (**b**) Arrhenius plot of the different regions of the ceramics after electroreduction. The extremely low activation energy of 0.012 eV accompanies the electrical transport process between 50 and 180 °C. At higher temperatures, the activation energy increases to 0.34 eV.

The optical inspection of the decolouration induced by the electrodegradation of the ceramic sample shows a regular change of all surface colours into dark grey (Figure 8a, left). After removing a 10 µm thick layer, the homogeneous grey discolouration disappeared, and an inhomogeneous electro-colouration in the form of islands (Figure 8a, right) became visible. This observation is essential for analysing the nature of the resistance’s electrical-driven reduction, showing the surface region’s preferential role in the degradation. After polishing, a dark area near the cathode can still be identified, indicating that this area was most heavily doped during electrodegradation. This strongest degradation (connected with decolouration) suggests that the removal of oxygen began at the cathode. The investigation mentioned above revealed that discolouration can be associated with the absorption of light on the free charges, which means that the electro-doping takes place preferentially in the “skin” of the ceramic.

Due to the reduced electric field in this bar sample, the final metallic stage of electrodegradation could not have been reached. Nevertheless, the analysis of the resistivity dependence as a function of the temperature of the different regions of the deoxidised sample was very useful for understanding the nature of the electrical transport in this degraded ceramic (Figure 8b). In the almost semi-metallic sample, at low temperatures, the E_a_ was only 0.012 eV. Using the data from our multi-probe system, we can show that the dependence of the local resistance in the different regions between the anode and cathode does not have the same activation energy. Moreover, both sections close to the anode and cathode significantly contribute to the total thermal dependence of the resistance change of the system Pt/PZT/Pt. In such a system, however, it is useless to determine a global value of the activation energy, because each section contains individual activation energy influenced by the electrodegradation strength.

**Figure 9:**
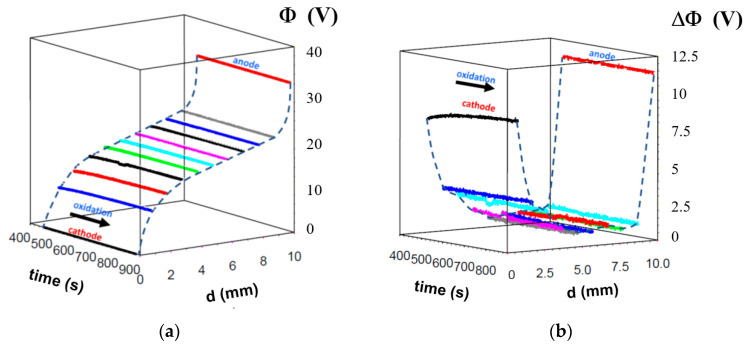
Exposure of an electrochemically-degraded PZT ceramic to high oxygen pressure at room temperature. (**a**) Local potential, and (**b**) the resistivity distribution as a function of exposure time. Different colors correspond to probe positions.

The application of the multi-probe analysis of the electrically-degraded sample’s reoxidation processes can contribute to understanding the kinetic incorporation of oxygen in such a system (according to reaction Equation (2)), primarily if the oxidation process is conducted at low temperatures (Figure 9).

(2) $$\frac{1}{2}O_{2}↓+Vö+2e^{\prime }\to O_{O}$$

This measurement is possible at low temperatures due to the low resistance of the electro-degraded ceramic. The reverse reaction to reduction, which can be observed when the deoxidised sample is exposed to a higher oxygen pressure, does not (as expected) increase the resistance of the entire specimen. Still, the selective increase in resistance starting from the cathode side, when the sample is electrically-polarised, can be observed.

This finding is also essential for analysing the nature of the deoxidation induced by the electric field, as it should be a “mirror image” of the reoxidation. The reversibility of the deoxidation allows for the classification of this process as a classical electrochemical redox process in the solid state.

### 2.5. Electrical Conductivity in the Nano-Scale of PZT Polycrystalline Thin Films

**Figure 10:**
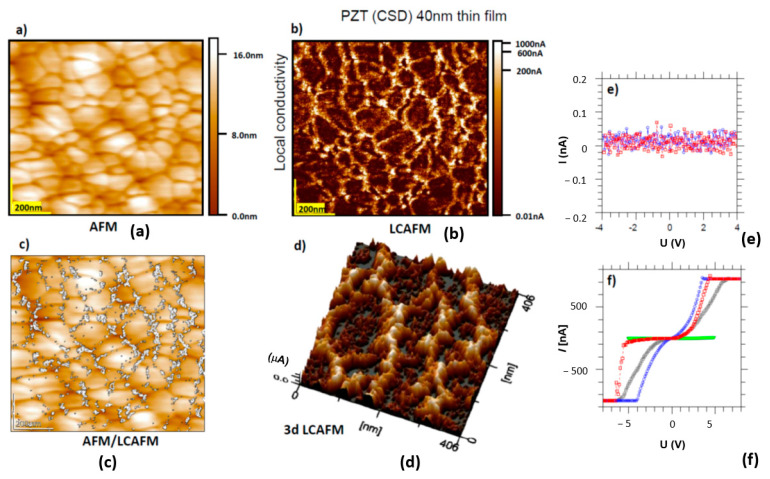
(**a**) Topography of the columnar PZT thin film; (**b**) LCAFM mapping of the conductivity of the stoichiometric layer; (**c**) overlap of the topography and LCAFM maps show the dominant contribution of the grain boundary to the electrical conductivity. High conductivity points are related to shorting the cantilever’s polarised tip or neighbouring filaments to the bottom electrode; (**d**) the 3D LCAFM mapping clearly shows the inhomogeneity of the electrical conductivity in the plane of the stoichiometric film. Note that the radius of the smallest conducting filament identified on the conducting maps is about 2 nm; (**e**) the I/U characteristics obtained for the grain; and (**f**) the collection of I/U curves showing the electrodegradation effect induced by a triangular voltage sweep of the filaments in the grain boundary. Multi-time polarisation leads to a successive increase in the local conductivity of the filament by several orders of magnitude [41,54].

Two limitations led us to investigate electrical conductivity at the nano-scale for thin films instead of solid ceramics. First, the high temperatures (350–450 °C) we used for the electrodegradation of ceramic PZT materials can destroy the Pt/Ir coating of the conductive cantilever. Second, at lower temperatures, the electric voltage at the tip of the cantilever should be extremely high (U > 1 kV), which can only be achieved in a specially-designed head of the LCAFM. For the analysis of the contribution of the grain and grain boundaries in the electrode gradation on the nano-scale, we used thin PZT with a columnar arrangement of grains. Due to the out-of-plane extension of the columns, one can present the current flowing along the grains and grain boundaries to the bottom electrode on the conductivity maps. Simultaneous scanning of the topography (Figure 10a) and local conductivity (Figure 10b) of CSD-PZT thin films, with a similar composition to the PIC PZT ceramics, allowed an enhancement of the local conductivity along the grain boundaries to be identified. The distribution of the conducting region in the columns’ boundary is discrete; some isolated or agglomerated filaments can be observed in this boundary. Similar to SrTiO_3_ crystals [41], the filaments can be electrically manipulated. In this manner, the filaments’ conductivity or their population can be changed by four to six orders of magnitude. The smallest filaments found on conductive maps (Figure 10d) are only 2 nm in diameter, corresponding to the typical radius of a single dislocation core in the ternary and binary oxides of transition metal atoms. It may be asked why a high concentration of conducting filaments can be detected on the conductivity maps of the dielectric PZT film with a bandgap of 3 eV. To understand this paradox, one can analyse the change in the I/U characteristics via repeated scanning of the same point on the boundary (Figure 10f). In contrast, the same type of repeated polarisation for the tip positioned on the grain does not affect the I/U characteristic (Figure 10e). This increase in the local conductivity of the filaments by electrically-polarised LCAFM tips with appropriately high voltages is an effect of *in operando* electrodegradation. If the polycrystalline film is under very low voltage, then the mapping only shows a current flow that corresponds to the noise of the voltage-to-current converter (10^−13^ A).

Scanning the same areas with a negative voltage in the range of 4–6 at the tip can transform the polycrystalline film along the grain boundary into regions with a high population of filaments of excellent conductivity. Although this process of electrically-induced switching of filaments can be preferentially observed at the grain boundary (Figure S1d), it should be noted that conductive filaments can also be found inside the grain. Still, their concentration and conductivity are much lower than at the grain boundary. The conductive filament inside the grain implies that the filaments must be galvanically connected to other filaments. This observation of the preferential transformation of filaments along the boundary suggests that this behaviour is very similar to the effect induced via the electrical addressing of individual dislocations in other transition metal oxides, for example, for the bi-crystalline boundary in SrTiO_3_ [41].

### 2.6. DFT Calculations and Source of the Charge Carriers in Electro-Degraded Ceramic

**Figure 11:**
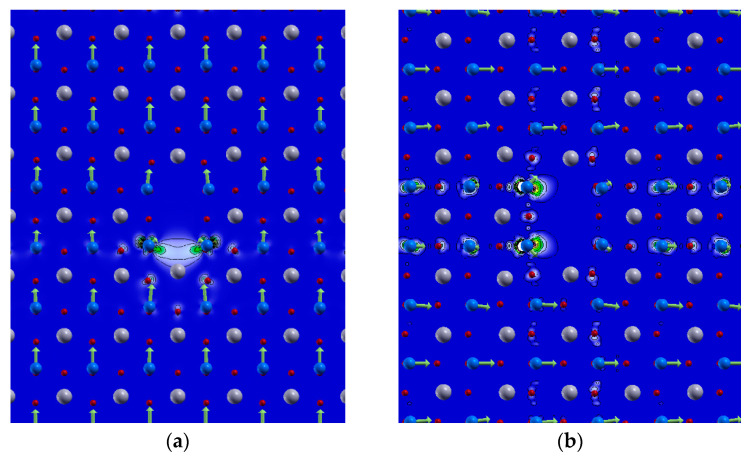
DFT calculations of the charge density of defect-induced states in PbTiO_3_ with O-Pb-O defect oriented (**a**) parallel, and (**b**) perpendicular to the polarisation. Pb atoms are shown in grey, Ti in blue, O in red, and green arrows marking local polarisation.

**Figure 12:**
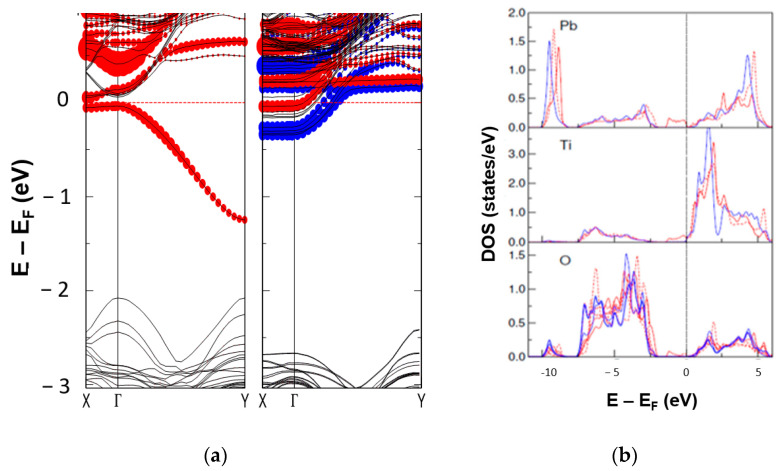
(**a**) Band structure of PbTiO_3_ with O−Pb−O defect oriented parallel to the polarisation (see Figure 11a) and perpendicular to the polarisation (Figure 11b), respectively. The size of the dots marks the weight of the states at Ti in the lower (**left**) part of the defect for the parallel (perpendicular) orientation. The red and blue colours in the middle panel show the different spin polarisations of the states; and (**b**) density of states (DOS) for the case with the defect parallel to the polarisation. The red and blue colours mark contributions from atoms around and far from the defect, respectively. The full and dashed red lines indicate DOS from atoms in the lower and upper parts of the defect.

The DFT calculations show the origin of the charge carriers appearing in the electro-degraded ceramic. To keep the results transparent on an atomic scale, we used PbTiO_3_ as a model system that shows structural and ferroelectric properties similar to PZT without explicitly speculating on the cation disorder. In the case of the poling field parallel to the orientation of the defect plane (Figure 11a), the two electrons left behind localise on one of the Ti pairs neighbouring the missing O atoms. This induces a highly dispersive defect state in the direction of the extended defect, which is 0.2 eV split off from the conduction band (Figure 12a). Based on the atomic positions per unit cell and assuming the valencies of Pb^2+^, Ti^4+^ and O^2−^, the polarisation is almost unperturbed locally and only diminishes due to the defect state. In contrast, when the poling is perpendicular to the O-Pb-O axis (Figure 11b), we obtain a spin-polarised defect state located at the two Ti atoms next to the two missing oxygen O rows. Such a weak magnetic response has already been observed in defected PZT single crystals [55]. The state is located at and in the conduction band minimum, making the system metallic (Figure 12). The polarisation breaks down in the rows perpendicular to the defect and its periodic copies in other unit cells.

## 3. Discussion

The numerous experiments described above permit discussion of a model of the electrically-driven transformation of PZT ceramic into metal-like conduction under law pressure (cosmic conditions). The model is based on the following points, which are discussed below and which can be drawn due to the simultaneous measurement of the electrodegradation and oxygen effusion:

1. Electrodegradation progression increases under reduced oxygen activity, as is the case under cosmic conditions;
2. Resistance change during electrodegradation under DC action is comparable to that observed in ternary oxides in the paraelectric phase, as it is in SrTiO_3_ or KTaO_3_ crystals [20]. A difference between them centres on the type of ions moving towards the cathode in the PIC ceramics, which creates a virtual anode. Based on the EDS mapping and XPS data (Figure S1e, we found that the concentration of Pb in the grain boundary did not increase. However, a reduction in PbO already occurs at 200 °C, and hence the lead in a metallic state was observed at 400 °C on the surface. This kind of electrodegradation is at a relatively low electric field of 100 V/cm. Hence, we state that these are the Pb cations that move into the cathode, creating the aforementioned virtual anode;
3. Through *in operando* measurements of the electric potential distribution, we showed that in a high electric field, the oxygen ions’ movement towards the anode predominates. In this process, the part of the sample lying between the cathode’s geometric and virtual positions is more conductive than the rest of the ceramic, and the maximal potential drop is localised at the virtual cathode. In contrast to the single crystal, the cathode front’s position is not sharp in the ceramic, and when the virtual cathode is close to the anode, maximum oxygen effusion occurs. The oxygen escape allows the electrodegradation process to be classified as electro-induced deoxidation or solid-state electrolysis [20];
4. The determination of the transfer number showed that the electric charge transport is a mixture of electrons and ions, but in all phases of the electrodegradation, the electrons predominate. Ionic transport disappears when metallic conductivity, in the last stage IV of electrodegradation, occurs;
5. The electrodegradation is an activated process described by the following current power law [5] I(t) = I_0_ × t^−n^, where t is the time and the n value is the function of the temperature. With increasing temperature, the exponent n becomes smaller, and so the idea of Sidebottom [56]—stating that the exponent of the power law decreases with decreasing dimensionality of the ionic conducting paths—can be applied to a percolation network of conducting dislocations in electro-degraded PZT (see Supplemental Materials S1). The LCAFM measurement and etching studies have shown the existence of the network of dislocations (conducting filaments) in the grain boundary of thin films. In particular, the dendrite-like fractal structure was proposed by Scott, et al. [15] to analyse the origin of the fatigue effect. An additional argument for the involvement of the mentioned network derives from the discolouration of the ceramics sample with the bar-like geometry. Due to a higher concentration of dislocations in the surface region generated during polishing, this region was mostly electro-degraded;
6. The extremely low oxygen outflow, leading to the insulator-metal transition, suggests that the regions (sources) of escaping oxygen are small and galvanically connected. Despite this, these regions are inhomogeneously distributed in the ceramic sample, which is reflected by non-uniform conductivity at the nano-scale;
7. The network of conducting filaments is created at the grain boundaries. The core of dislocations constitutes semiconducting nanowires with an additional d^1^ state of Ti and Zr, which follow the invariance of Burger’s vector and constitute a galvanic short circuit of the insulating grains. During the electrodegradation, the current flows through such a filament network. Hence, the deoxidation process is selective and preferentially reduced to the core of dislocations. Incorporating additional oxygen vacancies in such filaments can generate Ti and Zr states close to the bottom of the conduction band. Then, the filaments can switch into metallic nanowires, and this behaviour is responsible for the current power law;
8. Although we did not perform measurements of the local electric field near the virtual anode (e.g., through nano-potentiometric studies), we submit that these fields are extremely high. This agrees with the electric field-driven cold emission of electrons reported for PLZT ceramics above T_C_, which may even lead to PZT ceramics damage, as was observed [57]. The strength of the electric field locally reaches a value higher than 10^6^ V/cm and transforms the dislocation network into metallic filaments through destruction by hot electrons of the TiO_2_ or ZrO_2_ bonding in the vicinity of the virtual anode. The valence of Ti or Zr atoms is thereby reduced, and a new metallic state is created on the core of the electro-degraded network. Therefore, the metallic component of the network is extended.

**Figure 13:**
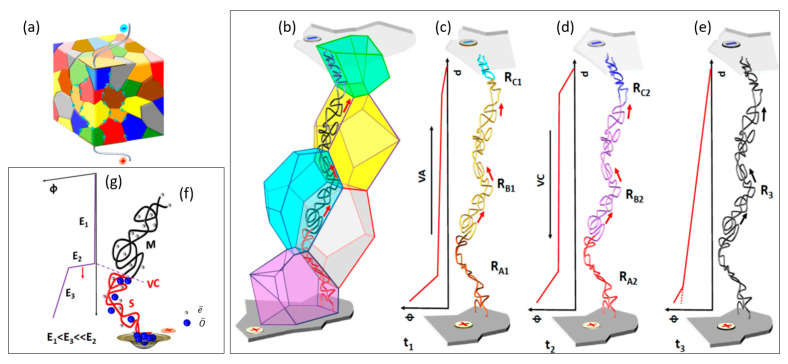
Scheme of (**a**) preferential current channelling along grain boundaries in an electro-degraded PZT ceramic; (**b**) dislocations along the grain boundary and different stages (**c**–**e**) of electrodegradation in time t_1_, t_2_ and t_3_. Φ is the potential, R the resistance, with the valence band virtual anode (VA), conductive band virtual cathode (VC), and d being the distance; (**f**) a schematic representation of the sequential transformation of two core dislocations into metallic filaments in the last stage of the electrodegradation; and (**g**) example of the potential Φ distribution along the metallic M and semiconducting S segments.

The processes accompanying the electrodegradation described above are graphically depicted in Figure 13. For low-voltage (Figure 13c), the initial parameters of the resistance of the three parts of the conductive path show the following relationship: R_A1_ > R_B1_ > R_C1_. *In operando* determined potential distribution suggests the moving of the virtual anode into the cathode (for details, see Figure 6b). Electrodegradation with a higher voltage (Figure 13d) started the longest part of the process. At the beginning of the process, the resistance close to the anode and cathode was similar to R_A2_ ≈ R_C2_ > R_B2_. In this stage, the resistance reduction was connected to the virtual cathode moving towards the anode (see Figure 6c). In the last stage (Figure 13e), the network of dislocations became metallic and short-circuited the electrodes with resistance R_3_. Note that despite the change in the relative ratio between the resistances in the different sections of the conductive path, the total resistance continuously decreased during electrodegradation, except for the metallic state. In the interface between filaments with metallic conductivity and semiconducting properties, a high potential drop exists (Figure 13g); it is at the actual position of the virtual cathode. The local field in this region is high enough for “cold electron” emissions. These electrons destroy single bonds between the oxygen and transition metal ions. In this reaction occurs the splitting of oxygen into octahedra, with free oxygen ions migrating into the anode. In the last phase, the oxygen effuses from the sample. Otherwise, if the anode is tight, it can deform the surface and blistering is observed.

For further analysis of the electrodegradation at the nano-scale, we would require PZT single crystals. Then, the SPM and TEM techniques would allow the physical chemical processes responsible for this inconvenience and deterioration of the physical properties of electro-degraded ceramic to be determined.

## 4. Materials and Methods

Sample characterisation: Most measurements were performed on the PZT piezo-ceramic with the commercial name PIC 151 (PI Ceramic, Germany). Its composition belongs to the PZT morphotropic phase boundary, with 2–3% of Pb(Ni_1/3_Sb_2/3_)O_3_ [58]. We checked this composition by means of EDS analysis. The nano-scale electrical conductivity was mapped in the PZT polycrystalline thin film deposited on a Pt electrode (110 texture) using the CSD technique.

Cosmic/vacuum and normal conditions: Establishing the true cosmic conditions in our vacuum chamber is impossible. However, the UHV conditions can effectively simulate the partial oxygen pressure in space. The vacuum conditions during the experiments corresponded to p_O_2__ = 0.02 mbar, whereas the normal conditions were p_O_2__ = 200 mbar.

Electrodegradation process measurements: Electrical measurements of the electrodegradation process of PIC ceramics were carried out for two different geometries of the samples. The so-called two-points technique was used for the specimens with the capacitor geometry—the typical geometry for the piezoelectric actuators. The classical bar geometry was then used to study the potential distribution in different stages of the electrodegradation process. The simultaneous measurement of the current and potential, which was performed via electrometric probes—positioned equidistantly on the surface between the anode and cathode—allowed analysis of the electrically-induced resistance R changes. In order to measure the potential distribution when applying a high voltage to a ceramic sample with a resistance of 1 TΩ, a multi-probe analysis system that we specifically developed for this purpose was used.

QMS and LCAFM measurements: Oxygen effusion from the specimen was identified and quantified using the QMS spectrometer e-Vision+ (Residual Gas Analyzer, 1–200 amu, with Electron Multiplier, Open Ion Source, MKS). Conductivity measurements (LCAFM) were made with a JEOL 4210 microscope under high vacuum conditions.

Raman spectroscopy: Raman micro-spectroscopy was performed using a 785 nm laser with a power of 100 mW. Spectra in reflectance were analysed using a HORIBA iHR320 spectrometer paired with a HORIBA Syncerity CCD-detector in 180° scattering geometry. The Raman data were rastered using a motorised table in x and y directions with a step size of 40 µm. At each position, a full spectrum was recorded.

DFT calculations: We employed density functional theory in the generalised gradient approximation proposed by Wu and Cohen [59], adding a Hubbard U on the Ti d and O p states to obtain a good description of the band gap and defect states. The calculations were performed with the full potential linearised augmented plane–wave method implemented in the Fleur code [https://www.flapw.de] (accessed on 6 March 2023). To simulate the extended O-Pb-O defect, we chose 4 × 5 × 1 unit cells. When the defect was aligned parallel to the polarisation in the y-direction, the dimensions were 4a × 5c × a (with a = 3.881 Å and c = 4.145 Å), and when the polarisation was along x and perpendicular to the defect, the dimensions were 4c × 5a × a. The 2 × 2 × 8 *k*-point mesh was used to sample the Brillouin zone, and the plane–wave cutoffs were 4.0 a.u.^−1^ and 15.1 a.u.^−1^ for the wave functions and charge density, respectively. The Hubbard U was U_d_ = 5.5 eV and J_d_ = 0.7 eV for Ti (compare [60]) and U_p_ = 8 eV for the O with muffin-tin radii of 1.75 a.u. and 1.5 a.u., respectively.

SEM measurements: The microstructure analysis of the ceramics was performed using a JSM-5410 scanning electron microscope (SEM) by Oxford Instruments with an energy dispersion X-ray spectrometer (EDS). The apparatus was equipped with an energy dispersion X-ray spectrometer with a Si(Li) X-ray detector. The vacuum in the test chamber was 10^−4^–10^−5^ Pa. The chemical composition was determined using ISIS- 300SEMQuant software. The measuring error was about 1 at %. Secondary electron (SEI) and backscattered electron (BEI) images were obtained for the polished surfaces of the PZT ceramics and the time-dependent etching process.

## 5. Conclusions

Due to the use of the PZT materials in cosmic applications, in which an enormous low activity of oxygen plays the main role, the fatigue effects became a factor increasing the induced electrodegradation (deoxidation). Pan, et al. [18] observed the electro-stimulated removal of oxygen, but under standard conditions; in a vacuum, the progression of the process is much higher. In studying the nature of the fatigue effect, we limited our investigation to the paraelectric phase focusing on essential mechanisms in defects and the electronic structure, which were responsible for the transition into a metallic state at an extremely low effusion of oxygen. The power law I(t) = I_0_·t^−n^ describes temperature-dependent electrodegradation. It could be related to the reduction of the fractal dimension in the hierarchical dislocation tree, the dimension of which decreases with increasing temperature. We conclude that the transition into a metallic state for 10^16^ oxygen atoms per 1 cm^3^ is connected to the creation of the nano-channels along the core of the dislocations localised along the grain boundaries. The investigation using the etch pit technique and LCAFM shows that the dislocations agglomerated at a grain boundary can change their electrical and mechanical properties by means of electrical stimuli. Our analysis has shown that the fatigue effect’s important mechanism is similar to the resistive switching in other model perovskite materials such as SrTiO_3_ and possesses many similar stages of the typical electrodegradation process. In contrast, in the first stage, the new aspect of this ceramic’s electrical transformation is the virtual cathode’s creation. As electromigration of the electrode materials was not observed, we state that it is connected with Pb ions. The possibility of the thermally-induced segregation of the Pb ions in the surface layer was proved by using *in operando* XPS studies. The last steps of the electro-induced deoxidation, further stimulated by Joule heating, led to the metallic short circuit of the PZT ceramic. This behaviour, which is identical to the prototype perovskite ternary oxides, suggested the preferential removal of oxygen from the core of dislocations and the creation of a d^1^ state close to the Fermi level, which exhibits the DFT calculation.

It was also shown that the restoration effect of insulating properties, called self-healing, via electrochemically-forced oxidation, is more effective than oxygen incorporation by a chemical gradient alone. Moreover, we have shown that the nature of the fatigue in the paraelectric phase is analogous to the classical resistive switching mechanism. However, the proposed destruction of the bonding between oxygen and transition metal ions, which can cause transformation into the metallic state, requires an additional potentiometric study at the nano-scale.

## Data Availability

Data can be provided upon request.

## Supplementary Materials

The following supporting information can be downloaded at: https://www.mdpi.com/article/10.3390/molecules28093652/s1. Supplemental Materials S1: Additional descriptions of experiments—Microscopic inspection (Figure S1a,b), Dielectric properties (Figure S1c), Dislocations in the PZT ceramics (Figure S1), surface characterisation of PZT thin film (Figure S1e–g), and Supplemental Materials S2: Additional descriptions of experiments by the multi-probe system (Figure S2a,b)—PDF file. References [61,62,63,64,65,66,67,68,69,70,71,72] are cited in the Supplementary materials.
