# Supplementary material for: The Electrodegradation Process in PZT Ceramics under Exposure to Cosmic Environmental Conditions
Source: Molecules. 2023 Apr 22;28(9):3652. doi: 10.3390/molecules28093652 (PMC10180167; doi:10.3390/molecules28093652)
Supplement: Supplementary file 1 [file molecules-28-03652-s001.zip › molecules-2299394-supplementary.pdf]

Supplement Materials S1

Microscopic inspection

Since the reduction and electrical degradation of ternary transition metal oxides with perovskite structure lead to the introduction of d^1 electrons into the system, a strong optical absorption [1] can be observed in a metallic channel coloured black (Figure S1a). This assumption about the local character of electro-degradation can explain the paradoxical situation with the I/M transition, which seems to occur at extremely low concentrations of d^1 electrons. Since the amount of emitted oxygen comes only from the channel (Figure S1b), the concentration in the channel is much higher. However, there were no deviations from the correct chemical composition for different sample regions (Figure S1a).

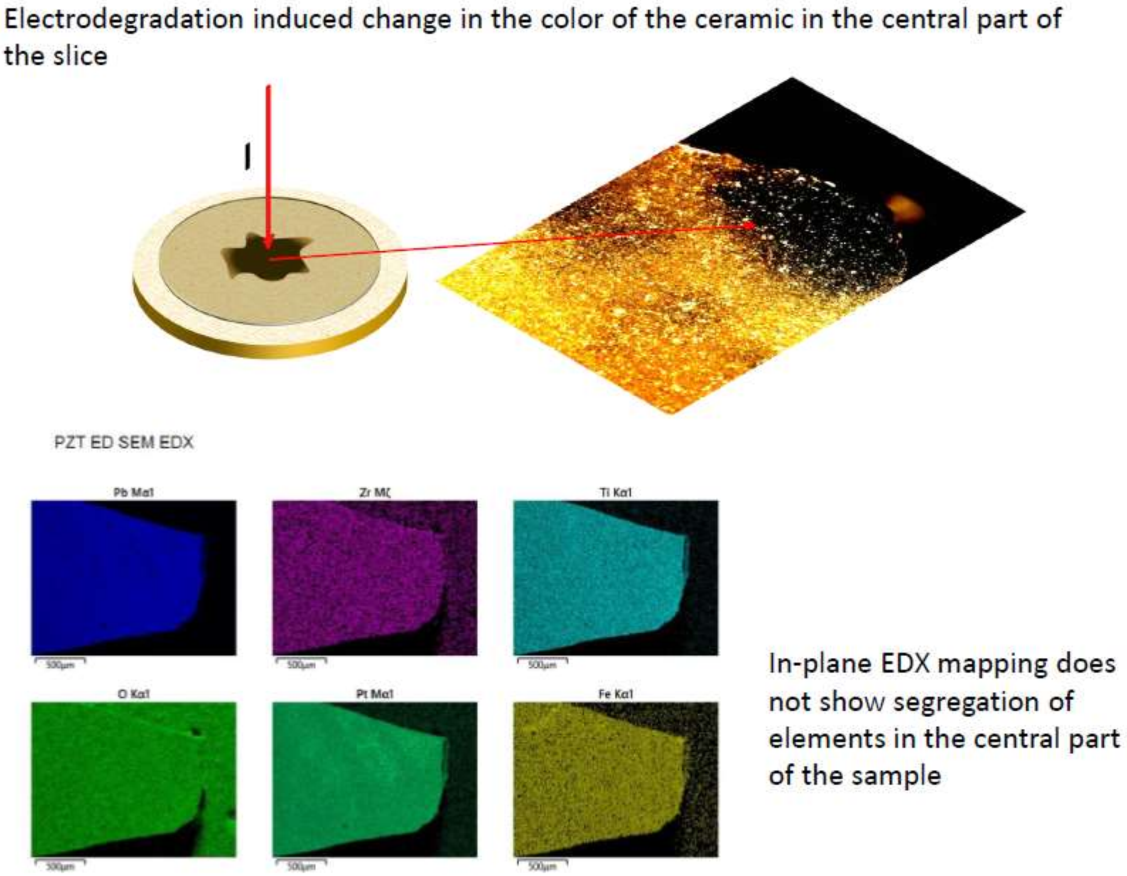

Figure S1a. Microscopic inspection of the fractured ceramic sample with metallic properties, performed for a specimen with typical capacitor geometry, shows that the change to dark colour is limited to only a tiny area, to a channel between the cathode and anode in the fractured surface.

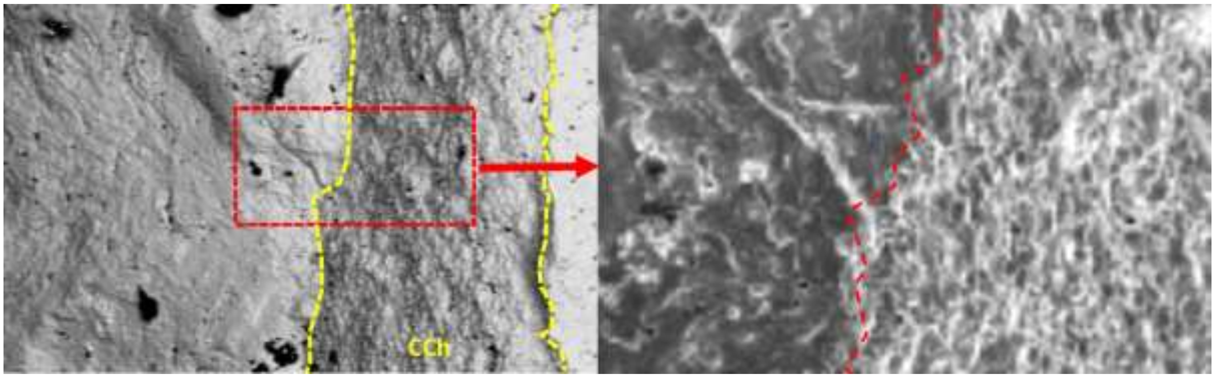

Figure S1b. Fractured surface from the region of electro-degraded ceramic with the conduction channel (CCh). One can distinguish the difference in the cleaving of the grain in the stoichiometric part of the ceramic and the conduction channel with dominant intergranular cleaving. The same observation was reported by Tan and He [2, 3].

Changes in the permittivity before and after the electro-degradation process, which reflect the processes described above, are presented in Figure S1c as a function of frequency. Ten-time decrease in the value of permittivity in the whole temperature region investigated can be recognised. Moreover, dielectric dispersion increases and takes a strongly diffused character, especially above the transition point. At the same time, the conductivity of electro-degraded ceramics became much higher than in the virgin sample.

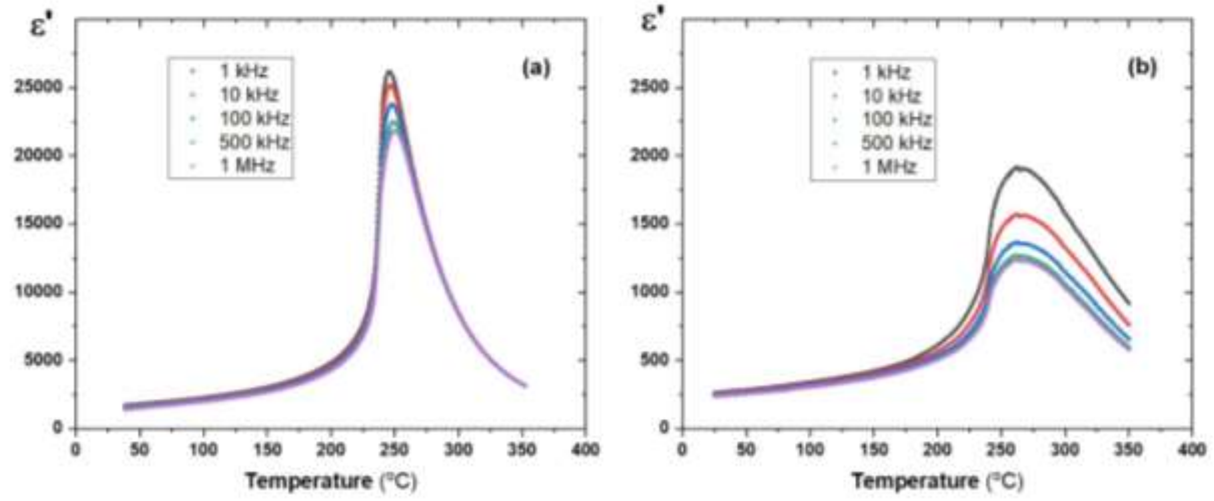

Figure S1c. Changes in the permittivity before (a) and after (b) electro-degradation process.

Note the difference in the scale of permittivity.

Dislocations in PZT ceramics

The extremal low effusion of oxygen (Figure 2a) and the investigation using LCAFM on the nano-scale (see section 2.5) suggest that the source for the oxygen could be only limited to the core of dislocations, which after removal of oxygen are, in fact, selectively

doped and in result contribute to the enhancement of the conductivity of the electro-reduced ceramic. The places of dislocations in cross-sections of ceramic are challenging to be observed using LCAFM. Due to complicated preparation, the TEM investigation observes the dislocations in a small ceramic segment possible. We have chosen chemical etching to investigate dislocations on the metallographically grinded surface. This simple but effective technique allows high-precision studies of the localisation of the dislocations which exist along the crystallographic boundary [4]. The correlation between the position of etch pits and conducting filaments supports the idea of preferred channelling of the current along dislocations. If this assumption is correct, then the character of the etch pits distributed in ceramics without and after electrodegradation should be observed as evident dissonance. The sample with conducting channel has been polished to conduct the experiment after electrodegradation. Using the SEM, chosen regions of the ceramic were observed as a function of the etch time (Figure S1d).

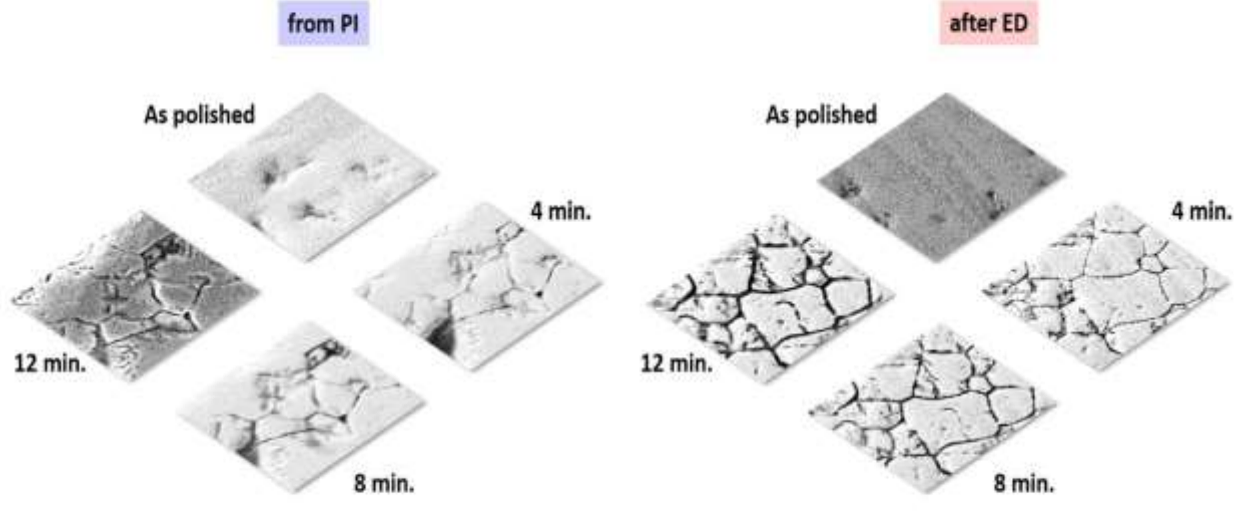

Figure S1d. Selective etching of grain boundary shows a higher progression of the protonation reaction for the boundary in the discoloured region (a conduction channel).

Although the starting roughnesses of both areas were the same, we observed a dramatic increase in the etched region of the conducting channel. A question arose, how can we correlate the local colouration and enhancement of the electrical conductivity with the chemistry of the etching. It is self-evident that the chemical attack on the core of dislocations and local stoichiometry changes in perovskite structure is much more effective than for the perfect matrix. Therefore it can be observed that etch pits possess the shape of an inverted pyramid. The mechanism of dislocation disclosure is as follows. The best result of a selective decoration of the end of dislocation lines for $SrTiO_3$ crystals was reached using HCl, HF or HNO_3 etchants at 80–90 ºC [4-10]. That is why the acid HNO_3 was used at 80 ºC for ceramics. The following chemical reaction of the protonation takes place:

$$\mathbf{PbZr_{1-x}Ti_xO_3 + 2HNO_3 \rightarrow Pb(NO_3)_2 + (1\text{-}x)H_2ZrO_3 + xH_2TiO_3 \rightarrow Pb(NO_3)_2\downarrow + (1\text{-}x)ZrO_2\downarrow + xTiO_2\downarrow + H_2O}$$

After the electrodegradation, the depth of the etching pits decorating the grain boundaries is much greater (Figure S1d). This proves that the structure of the grain boundaries possesses a higher concentration of oxygen vacancies [11-13] which increases the number of dangling bonds and protonation of $PbZrO_3$ and $PbTiO_3$ occurs. Protonation is an exchange reaction that introduces protons in place of cations. Protons are substituted for lead, and the H_2ZrO_3 or H_2TiO_3 system is formed. Such solid protic acids are unstable and immediately dissolve; this is where a hole is created. In other words, this is in the lattice with fewer lead ions; therefore, it is easier to get cation exchange (protonation).

Surface characterisation of PZT thin film

The analysis of the point defect chemistry of PTO, PZO and PZT materials are based on the assumption that the defect can be described in terms of the Schottky disorder. For

such a defect model, the surface should play a special role. It is an essential interface between the solid (here, the ceramic or thin films) and the environment, where the dependence on the activity of the gases, with the high pressure in the equilibrium state, can create or annihilate the point defects. This region can be easily analysed with surface-sensitive methods, e.g., XPS. Because the centre of our interest is the study of the electrodegradation (electro-deoxidation), the UHV conditions and high temperature give the possibility in situ and *in operando* to analyse the electronic structure.

As expected, the measurement of the *ex-situ* prepared sample showed substantial contamination of its surface. The O1s and C1s line analysis showed the compounds with higher binding energy, typical for the physisorption (for O1s) and chemisorption of water and hydroxyl groups. Despite this surface covering with the untypical element of the matrix of PZT, the Ti2p, Zr3d, and Pb4f core lines consist of only single doublets without additional peaks. *In operando*, the XPS spectroscopy showed that the temperature increase leads to the subsequent desorption of water at 200 ºC (see O1s spectra) and removing of CO_2 at 400 ºC (Figure S1e).

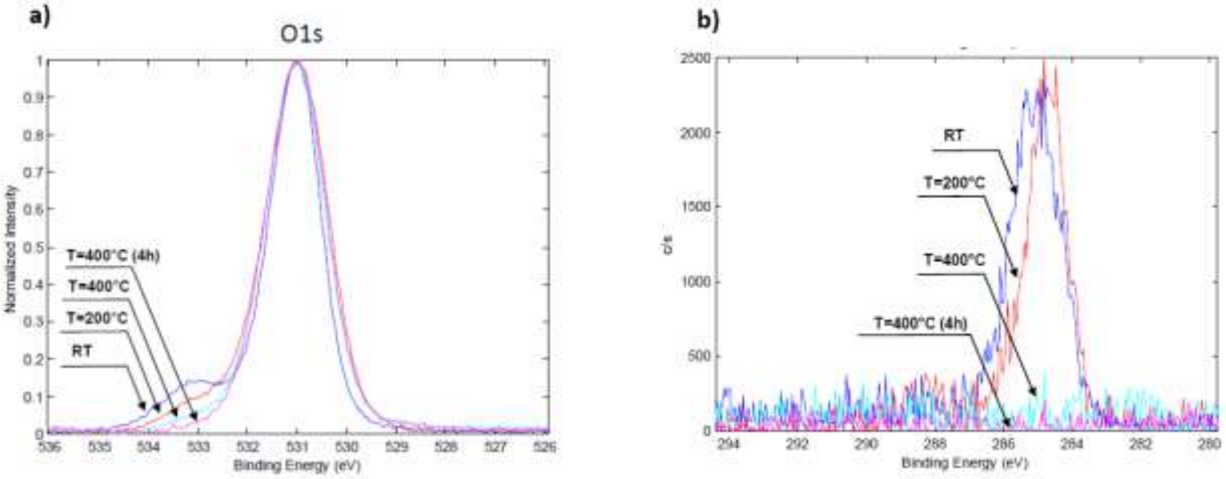

Figure S1e. a) Changes of O1s and b) C1s core lines of thin PZT films induced by thermal treatment under UHV conditions. The XPS measurements were made *in operando*.

This founding confirmed the correctness of the choice of thermal conditions for electrodegradation because the sample was clean for such a temperature. In this way, we can omit analysis of degradation models that consider the presence of water. In this case, the critical influence of water as a potential "accelerator" of the cation migration does not play a significant role. XPS allows studying the binding energy and determining, depending on the angle, the local composition in a 4-6 nm thin layer. If the redox process starts from the surface, then we should, on the one hand, observe the change in valencies of elements in the matrix, or, on the other hand, the evolution of the local stoichiometry should take place. At 400 ºC, the O1s singlet and Ti2p and Zr3d doublets can be observed, except for the Pb4f line (Figure S1f). Two modifications can be observed in such a doublet: the first one is the reduction in the relative intensity, which suggests depletion of the concentration of Pb in the matrix. The second one is connected with a new compound on the Pb4f core line with lower binding energy, typical for a metallic PbO electronic state. Because measurements of PZT *in operando* allow unequivocal identification of the actual electronic structure of the surface (without a modification coming from *ex situ* treatment), we state that we have to do with a *gentle reduction* of the Pb atoms into the metallic state. The increase in the temperature to 550 ºC and 650 ºC in the XPS spectrometer led to a more substantial decrease in the Pb stoichiometry and created a higher concentration of metallic Pb (Figure S1g). It is not surprising because the phase diagram for Pb and its oxides shows a similar tendency under low oxygen activity [14]. From the XPS data, one can draw another conclusion from the VB density of the state. Namely, because the VB region for contamination-free surface shows the same shape, the Pb state does not play an essential role in hybridising the O2p and d states of Ti and Zr.

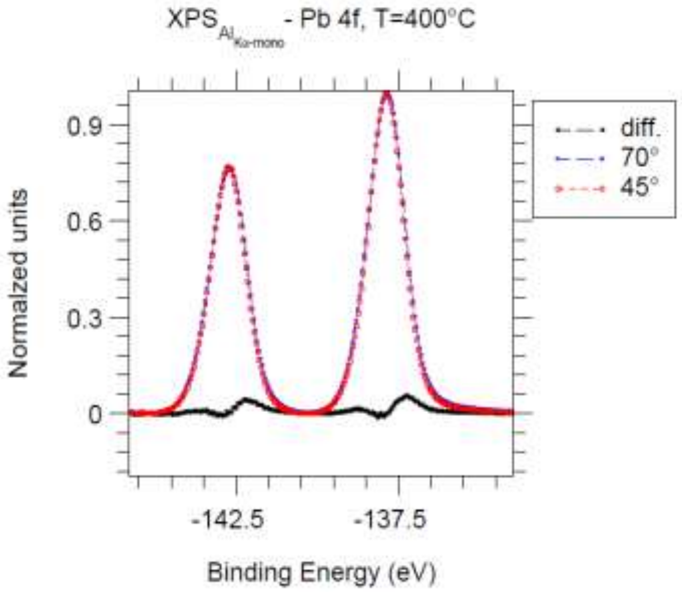

Figure S1f. Comparing the Pb4f core lines obtained at 45º and 70º (grazing incidents) shows the existence of doublets with typical binding energy for metallic lead. Due to the subtle differences between the XPS spectra, we did not do a line deconvolution. Instead, we used subtraction which distinctly shows the difference between the lines.

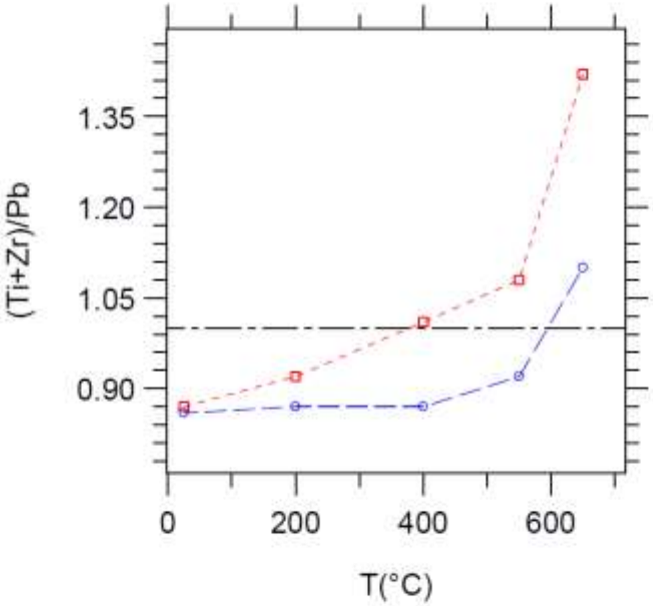

Figure S1g. Using the MultiPak software from Physical Electronics, the relative change between the stoichiometry of the BO_2 (B=Ti/Zr) and PbO sublattice was determined for

each temperature. XPS measurement was obtained *in operando*. The ratio between the atomic concentration of Ti and Zr relative to the Pb, for the surface layer detected at 70º (blue), indicates the beginning of PbO segregation at 500 ºC. The stoichiometry analysis for lower angles 45º (red) demonstrates that this process already starts at 200 ºC.

Supplement Materials 2

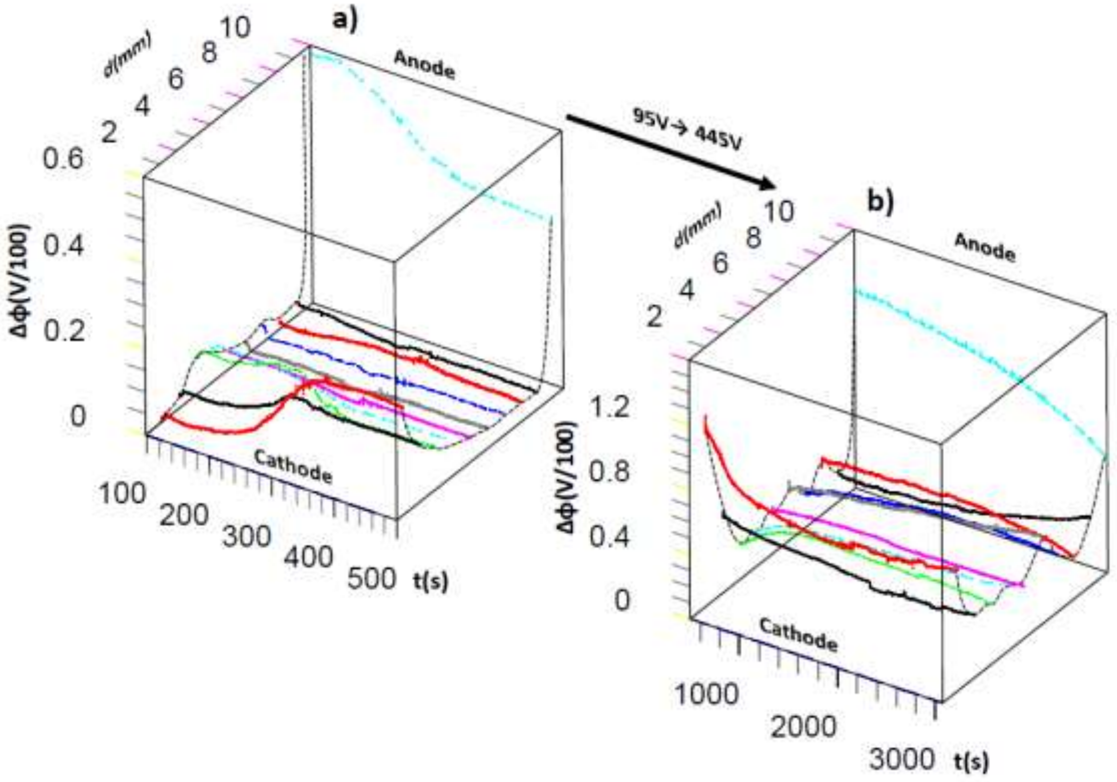

Figure S2a. Based on the data in Figure 6b, the local potential drop can be determined in all regions, with a 1mm length, between the cathode and anode, for two different polarisation voltages, a) 95 V and b) 445 V.

Based on the time-synchronised data acquisition by the multiprobe system (Figure 5), we can analyse the resistance in all regions between the anode and cathode. The derivation of the local potential drop (Figure S2a) from the "global" potential distribution (Figure 6), local resistance (Figure 7) and the local power load (Figure S2b) allows not only the calculation of the local electrical field but shows the progression of the resistance

degradation in regions between anode and cathode. In the last stage of the electrodegradation, the potential drops close to both electrodes show only small differences relative to the voltage in the rest of the sample's regions (Figure S2a).

The switching on the higher voltage 445 V lead to symmetrisation of both potential drops close to electrodes (Figure S2a b); the electric field in other regions is 3-4 times lower. The electric field's local strength near the electrodes reaches the value of 1 kV/cm and is much higher than in the other regions of the sample. This calculation is based on the position of the last electrode at a distance of 1 mm from the anode. The measurements of the potential distribution on single crystals with a moving probe show that the potential drop close to the anode takes a much smaller length[7]. Therefore, the local electric field is much higher. Indeed, potentiometric measurements on the cross-section Pt/STO confirmed that the local electric field could reach the value of 0.1 MV/cm. In the last stage of the electrodegradation, the potential drops close to both electrodes, showing only small differences relative to the voltage in the rest of the sample's regions [7].

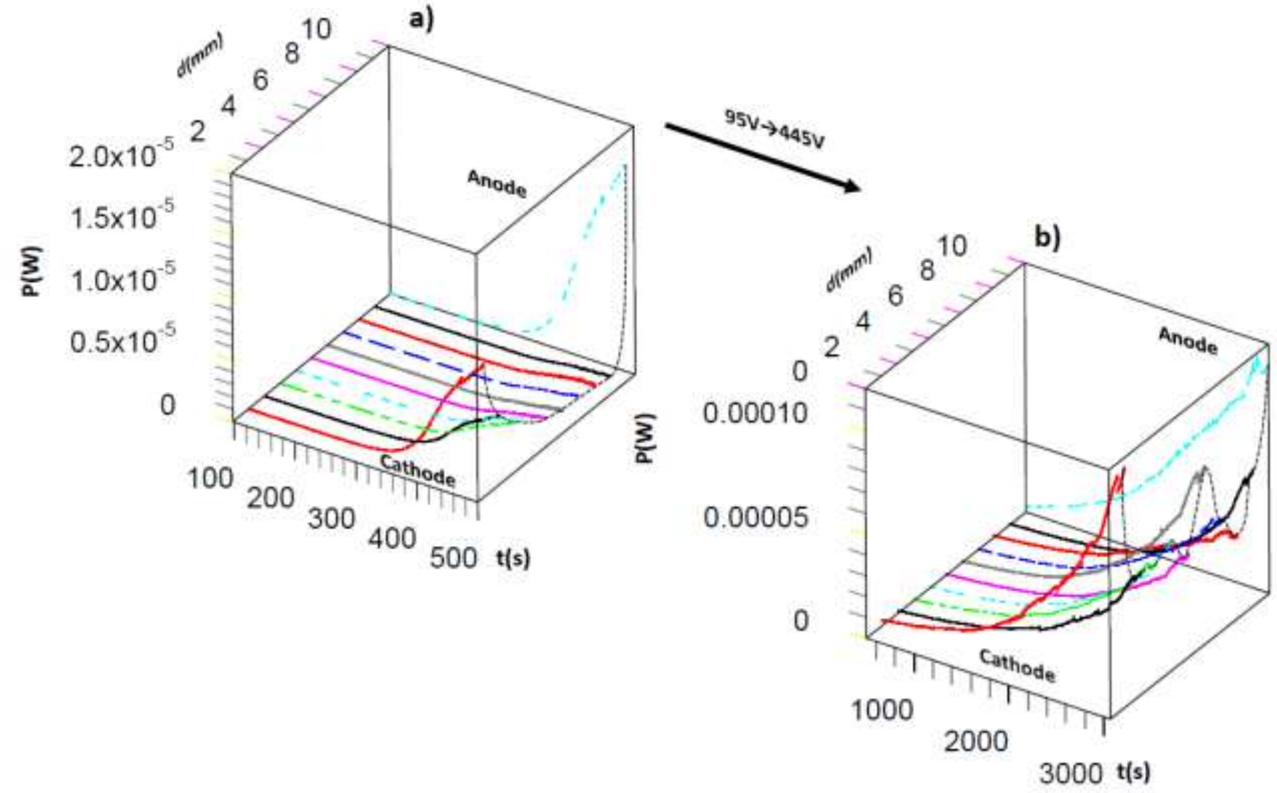

Figure S2b. a) Local power losses along the ceramic PIC sample during the electrodegradation for low voltage showed that the regions close to the cathode and anode contributed significantly to the losses. b) In contrast, the polarisation with high voltage changes the distribution of power losses by increasing the electrodegradation time blurring the difference in the dominant power load of the two interfaces close to the electrodes. Due to the linearisation of the potential distribution in this stage (see Figure7b on the left in the main text), the power losses are homogeneous.

Based on the time-synchronised data acquisition from the current flow (Figure 6a) and local potential drop (Figure S2a), we can analyse the progression of resistance-degradation in all regions between the anode and cathode. It should be mentioned that the measurements of the electrodegradation with the multiprobe system did not reveal the step-like changes of the resistance (stage IV), which originated from the positive feedback between the Joule-heating and the local electric field action (see Figure S2b). In

the last phase of the electrodegradation (in the areas in the centre of the sample), the potential drops are not precisely the same. However, with a homogeneous ceramic object and uniform positioning of the probes on the surface, similar potential drop values can be intuitively expected. This observation puzzled us, because the verification of the correctness of the electrometric measurement confirmed that the interfacial resistance between electrometric probes and the surface of the sample in all phases of the measurement was several orders smaller than the input resistance of the electrometer. Therefore, it could not explain these fluctuations. Presented information allows for the analysis of the degradation from a new perspective.

This slow rate of electrodegradation, without typical sudden changes in resistance (see Figure 1), can be better understood if local electric losses in different regions are involved in the analysis. Despite the complicated distribution of local electrical losses along the probe at various stages of electrodegradation, the maximum values reached near the two electrodes are less than 0.1 mW. But our studies of the electrodegradation processes for different temperatures have shown that the necessary power value, which leads to the positive thermal feedback, is a few dozen mW.

References

[1] Rodenbücher, C., Menzel, S., Wrana, D., Gensch, T., Korte, C., Krok, F. and Szot, K. Current channeling along extended defects during electroreduction of SrTiO3. *Scientific reports*, 9, 1 (2019), 1-9.
[2] Tan, X., He, H. and Shang, J.-K. In situ transmission electron microscopy studies of electric-field-induced phenomena in ferroelectrics. *Journal of materials research*, 20, 7 (2005), 1641-1653.
[3] Weaver, P., Cain, M., Stewart, M., Anson, A., Franks, J., Lipscomb, I., McBride, J., Zheng, D. and Swingler, J. The effects of porosity, electrode and barrier materials on the conductivity of piezoelectric ceramics in high humidity and dc electric field. *Smart materials and structures*, 21, 4 (2012), 045012.
[4] Szot, K., Rodenbücher, C., Bihlmayer, G., Speier, W., Ishikawa, R., Shibata, N. and Ikuhara, Y. Influence of dislocations in transition metal oxides on selected physical and chemical properties. *Crystals*, 8, 6 (2018), 241.
[5] Paladino, A., Rubin, L. and Waugh, J. Oxygen ion diffusion in single crystal SrTiO3. *Journal of Physics and Chemistry of Solids*, 26, 2 (1965), 391-397.

[6] Szot, K., Speier, W., Carius, R., Zastrow, U. and Beyer, W. Localized metallic conductivity and self-healing during thermal reduction of SrTiO 3. *Physical review letters*, 88, 7 (2002), 075508.
[7] Szot, K., Bihlmayer, G. and Speier, W. *Nature of the resistive switching phenomena in TiO2 and SrTiO3: origin of the reversible insulator–metal transition*. Elsevier, City, 2014.
[8] Waugh, J., Paladino, A., DiBenedetto, B. and Wantman, R. Effect of Dislocations on Oxidation and Reduction of Single‐Crystal SrTiO3. *Journal of the American Ceramic Society*, 46, 1 (1963), 60-60.
[9] Rhodes, W. Etching and Chemical Polishing of Single‐Crystal SrTiO3. *Journal of the American Ceramic Society*, 49, 2 (1966), 110-112.
[10] Spalding, G., Murphy, W., Davidsmeier, T. and Elenewski, J. Faceting of single-crystal SrTiO3 during wet chemical etching. *MRS Online Proceedings Library (OPL)*, 587 (1999).
[11] Hensling, F. V., Du, H., Raab, N., Jia, C.-L., Mayer, J. and Dittmann, R. Engineering antiphase boundaries in epitaxial SrTiO (2019).
[12] Marrocchelli, D., Sun, L. and Yildiz, B. Dislocations in SrTiO3: Easy to reduce but not so fast for oxygen transport. *Journal of the American Chemical Society*, 137, 14 (2015), 4735-4748.
[13] Navickas, E., Chen, Y., Lu, Q., Wallisch, W., Huber, T. M., Bernardi, J., Sto¨ger-Pollach, M., Friedbacher, G., Hutter, H. and Yildiz, B. Dislocations accelerate oxygen ion diffusion in La0. 8Sr0. 2MnO3 epitaxial thin films. *ACS nano*, 11, 11 (2017), 11475-11487.
[14] Lamoreaux, R., Hildenbrand, D. and Brewer, L. High‐Temperature Vaporization Behavior of Oxides II. Oxides of Be, Mg, Ca, Sr, Ba, B, Al, Ga, In, Tl, Si, Ge, Sn, Pb, Zn, Cd, and Hg. *Journal of physical and chemical reference data*, 16, 3 (1987), 419-443.
